# GRAD: A Two-Stage Algorithm for Resolving Diagnostic Uncertainty in the Plasma p-tau217 Gray Zone

**DOI:** 10.64898/2026.02.03.26345302

**Published:** 2026-02-09

**Authors:** Harthik Parankusham, Eashwar Krishna

**Affiliations:** 1 Department of Physiology & Neurobiology, University of Connecticut; 2 Department of Molecular & Cell Biology, University of Connecticut; 3 Syntropi AI Group

**Keywords:** Plasma biomarkers, p-tau217, Alzheimer’s disease, machine learning, amyloid PET, diagnostic algorithm, gray zone, multimodal biomarkers, MRI volumetrics, health economics, clinical decision support

## Abstract

**Introduction:**

Phosphorylated tau-217 (p-tau 217) is widely used as a plasma-based biomarker for Alzheimer’s Disease (AD) detection, demonstrating superior accuracy for detecting brain amyloid pathology. However, 30–50% of patients fall within an intermediate diagnostic “gray zone” where biomarker results are indeterminate, often decreasing physician confidence and requiring subsequent diagnostic workup. To address this, we developed a two-stage machine learning algorithm ‘GRAD: Gatekeeper & Reflex for Alzheimer’s Disease’ to increase clinical confidence and reduce the AD health economic burden.

**Methods:**

We initially analyzed 320 participants from the Alzheimer’s Disease Neuroimaging Initiative (ADNI) with plasma biomarkers and amyloid PET. We then built a two-stage machine learning classifier mimicking real clinical workflow where the stage 1 ‘Gatekeeper’ used the gold-standard marker: p-tau217 with respective 25%/75% probability thresholds. The stage 2 ‘Reflex’ step applied Random Forest multi-marker classification (p-tau 217, AB42/40, NFL, GFAP) for difficult-to-diagnose gray zone cases. To ensure statistical robustness, leave-one-out cross-validation with bootstrap confidence intervals was used. We externally validated the GRAD architecture on 1,644 A4 Study participants, with MRI enhancement analysis in 1,044 gray zone cases. To measure cost-effectiveness we compared our GRAD-staged testing to universal PET.

**Results:**

The model’s ‘Gatekeeper’ resolved 55.6% of ADNI cases with 88.8% accuracy (NPV 91.8%, PPV 85.0%). The complete pipeline achieved AUC 0.867 (95% CI: 0.825–0.904), with 80.6% sensitivity, 80.0% specificity, LR+ 4.03, LR− 0.24. For the difficult-to-diagnose gray zone cases, the ‘Reflex’ machine learning model achieved AUC 0.755. In our A4 validation, the predictions correlated strongly with centiloid (r= 0.693). Expanding beyond plasma biomarkers, MRI integration improved gray zone classification from AUC 0.829 to 0.853 (p=0.014). The cost modeling analysis projected a 67% reduction in spending versus the current standard of universal PET.

**Discussion:**

Our clinically-staged diagnostic algorithm, ‘GRAD’, provides actionable classifications for the majority of patients while routing uncertain cases for additional workup. The GRAD framework offers a practical, cost-effective approach for implementing plasma biomarkers in clinical practice. Future iterations of this framework, with integration of novel biomarkers like MTBR-tau243 present a significant opportunity to alleviate the AD health-economic burden and eliminate expensive but unnecessary diagnostic measures.

## 1. Introduction

Blood-based biomarkers for Alzheimer’s disease (AD) enable detection of cerebral amyloid pathology without the cost, invasiveness, and limited accessibility of amyloid PET or cerebrospinal fluid analysis [1–3]. Plasma phosphorylated tau at threonine-217 (p-tau217) has emerged as the most promising candidate, with area-under-the-curve (AUC) values exceeding 0.90 for discriminating amyloid-positive from amyloid-negative individuals [4–6].

However, these impressive results require careful interpretation. Studies reporting AUC >0.90 typically evaluate cognitively normal individuals versus established AD dementia, populations with maximal biomarker separation. In clinical practice, the critical decision point occurs in patients with mild cognitive impairment (MCI), where substantial biomarker overlap exists without the stark clarity akin the patients typically used in existing studies. In typical populations, 30–50% of individuals fall within a “gray zone” where diagnostic certainty is insufficient for clinical decisions [7,8].

This gray zone problem has solidified the importance of therapy approvals with disease-modifying potential. The FDA label for lecanemab requires amyloid confirmation, and misclassification carries significant consequences: false positives expose patients to amyloid-related imaging abnormalities (ARIA) risks without benefit, while false negatives deny therapy to eligible patients [10,11].

We hypothesized that a two-staged diagnostic approach similar to existing reflex testing mechanisms in laboratory medicine could efficiently resolve gray zone uncertainty. A simple univariate screen (Gatekeeper) first identifies high-confidence cases, while a multi-marker classifier (Reflex) addresses uncertain cases.

Study objectives were to: (1) develop and validate a two-stage algorithm using Alzheimer’s Disease Neuroimaging Initiative (ADNI) data; (2) externally validate the model in the A4 Study; (3) evaluate MRI volumetric integration for gray zone cases; and (4) model health economic implications.

## 2. Methods

### 2.1 Study Populations

A cohort of 320 participants from the UPENN/Janssen platform with plasma biomarkers and amyloid PET scans were included in the ADNI Development cohort, including cognitively normal (44.4%), MCI (39.4%), and AD dementia (16.2%) participants. This was an intentional selection to reflect clinical populations where diagnostic uncertainty may exist. Amyloid positivity was defined as florbetapir SUVR >1.11 [12]; overall amyloid positivity was 48.4%.

For the external validation, 1,644 participants with plasma biomarkers (Lilly p-tau217, Roche GFAP/NfL) were included from the A4 study, which originally enrolled cognitively normal adults aged 65–85 with elevated amyloid (Centiloid ≥20) [13]. An additional 1,044 participants had MRI data for enhancement analysis.

### 2.2 Biomarker Harmonization

All biomarkers were Z-normalized to a reference population [14]; these consisted of cognitively normal, amyloid-negative individuals within each cohort. Values were log-transformed, then Z-scores computed as: Z = (log(x) - μ_ref) / σ_ref; this process anchors the values to a biological baseline.

### 2.3 Two-Stage Algorithm

For Stage 1 (Gatekeeper), a univariate logistic regression was run using pTau217_Z with probability thresholds of amyloid negative (P < 0.25, high confidence), amyloid positive (P > 0.75, high confidence), and finally the gray zone (0.25 ≤ P ≤ 0.75), which were routed to Stage 2 of the workflow.

For Stage 2 (Reflex), Random Forest classifiers (n_trees=100, max_depth=5) with balanced class weights were applied to the aforementioned gray zone cases. Features included primary biomarkers (p-Tau217, GFAP, NfL), statistically-generated features capturing biological relationships (tau_ab42_diff, gfap_tau_interaction), and demographic/genetic factors (AGE_Z, APOE4_carrier). For A4 gray zone analysis, hippocampal and entorhinal volumes (ICV-normalized, FreeSurfer-derived) were added to Reflex features.

### 2.4 Validation Strategy

Our internal validation method was leave-one-out cross-validation (LOOCV) with harmonization parameters recalculated within each fold; this prevented data leakage. The bootstrap confidence intervals (1,000 iterations) were calculated for all the associated metrics. More importantly, we developed an external validation procedure which applied the ADNI-trained model to the A4 trials after cross-platform harmonization. Given A4’s amyloid-positive-only enrollment, we evaluated correlation with continuous centiloid rather than traditional sensitivity/specificity measures. The statistical analysis consisted of AUC with DeLong confidence intervals, accuracy, sensitivity, specificity, PPV, NPV, likelihood ratios (LR+, LR−), and Brier scores. The MRI-included model comparison used DeLong’s test [15] and paired bootstrap.

### 2.5 Health Economic Modeling

To quantify GRAD’s health economic impact, we developed a decision-analytic model comparing four diagnostic strategies for a projected cohort of 10,000 patients: Universal PET ($3,000 per scan), Plasma + PET for the gray zone ($500 plasma + PET for 44.4%), our GRAD staged algorithm ($500 plasma + $200 reflex panel + PET for residual ~13%), and Staged + MRI (assuming MRI already obtained). It is important to note that universal assumptions were made, including a Gatekeeper resolution of 55.6%, Reflex resolution of 70% of the gray zone, and MRI-enhanced resolution of 80% of the gray zone. The first two parameters were derived directly from the ADNI LOOCV results (Sections 3.3 and 3.4, respectively), while the MRI-enhanced estimate is extrapolated from the classification improvement observed in the A4 gray zone analysis (ΔAUC = +0.025, p = 0.014; Section 3.7) and is consistent with evidence that hippocampal volumetrics independently predict amyloid status [25]. The unit costs reflect 2024 U.S. Medicare reimbursement schedules [29].

## 3. Results

### 3.1 Participant Characteristics

Table 1 presents baseline characteristics of the participants. The ADNI development cohort had a mean age of 72.5 +/− 6.8 years, was 47.5% female, and 89.1% White. 39.0% of participants were APOE ε4 carriers and the median p-tau217 level was 0.110 pg/mL. In comparison, A4 cohort had a mean age 71.8 +/− 4.7 years, was 58.0% female; 36.4% were APOE ε4 carriers and the median p-tau217 level was 0.152 pg/mL.

### 3.2 Understanding Algorithm Performance in Context

The overall AUC of 0.867 observed in this study is strong when compared against published literature. Studies reporting AUC >0.90 typically evaluate cognitively normal participants versus established AD-dementia patients; in such studies the maximal biomarker separation indicates optimal conditions that may not consistently exist in reality

Our ADNI cohort includes MCI patients, the key clinical decision point where uncertainty exists. When stratified by cognitive status, key AUC differences were observed: CN vs AD dementia (0.94); MCI cases alone (0.82); mixed cohort (0.867). This demonstrates utility in uncertain situations where the algorithm provides real value. The difference in results from studies utilizing research cohorts with highly restrictive exclusion criteria is not a limitation but rather a more honest representation of real-world performance.

### 3.3 Stage 1: Gatekeeper Performance

The Gatekeeper resolved 178/320 cases (55.6%) with 88.8% accuracy (95% CI: 83.4–93.1%). 98 cases were classified as amyloid negative (NPV 91.8%, 95% CI: 84.5–96.4%), 80 cases were classified amyloid positive (PPV 85.0%, 95% CI: 75.3–92.0%), and 142 cases were in the gray zone (44.4%) which were routed to Stage 2.

The 55.6% resolution rate is intentional by design. The algorithm identifies cases where p-tau217 alone is capable of providing a “High Confidence” classification, routing inherently difficult cases to the multi-marker Reflex model.

### 3.4 Stage 2: Reflex Performance

The Reflex model for gray zone participants (n=142) achieved an AUC of 0.755 (95% CI: 0.673–0.837), with an accuracy of 69.7%, a sensitivity of 74.0%, and a specificity 64.6%.

The most significant feature importances, shown as variables, were pTau217 (28.3%), tau_ab42_diff (14.7%), gfap_tau_interaction (13.2%), AGE_Z (10.8%) and APOE4_carrier status (8.0%). The engineered tau_ab42_diff feature which captured the inverse relationship between tau phosphorylation and amyloid clearance provided substantial predictive value beyond the individual markers. The Reflex improves gray zone classification from mere chance (50%) to 69.7% accuracy, which is meaningful for cases that by definition lack clear biomarker separation.

### 3.5 Overall Pipeline Performance

Table 2, shown below, presents the complete GRAD pipeline result metrics. Overall the AUC was 0.867 (95% CI: 0.825–0.904), accuracy was 80.3% (95% CI: 75.9–84.4%), sensitivity was 80.6% (95% CI: 74.5–86.6%), specificity was 80.0% (95% CI: 73.5–85.4%), PPV was 79.1%, NPV was 81.5%, LR+ was 4.03, LR− was 0.24, and the Brier score was 0.148.

At clinically relevant operating points, the model’s 90% sensitivity threshold yielded 62.4% specificity (LR− 0.16) while the same 90% specificity threshold yielded 68.4% sensitivity (LR+ 6.84).

### 3.6 External Validation (A4 Study)

The GRAD model predictions strongly correlated with centiloid (r= 0.693, p<0.001). We analyzed the sensitivity by amyloid burden, resulting in the following metrics: low centiloid (20–30) 18.4%, moderate (30–60) 32.8%, high (60–80) 42.1%, very high (>80) 75.4%. This correlation validates that predictions track biologically meaningful amyloid variation despite cross-platform application.

### 3.7 MRI Enhancement Analysis

Amongst the 1,044 A4 gray zone participants with a T-1 weighted MRI we found that the plasma-only AUC was 0.829 (95% CI: 0.812–0.846), the MRI-only AUC was 0.721 whereas plasma + MRI AUC was 0.853 (95% CI: 0.838–0.868).The observed improvement was statistically significant (ΔAUC=+0.025, DeLong p=0.014, bootstrap 95% CI [0.006–0.039]) and 99.7% of 1,000 iterations favored the MRI-enhanced GRAD model.

The hippocampal volume feature contributed 16.9% to the aggregated feature importance. Furthermore, stratification by hippocampal tertile revealed significant differences: small hippocampus demonstrated 74.7% amyloid positivity, with medium hippocampus at 57.5% positivity and large at 30.2% positivity - this was a 2.5-fold variation despite relatively similar pTau217 levels (0.146–0.170 pg/mL). This subsequent analysis for GRAD demonstrated that MRI provides independent diagnostic information that cannot captured by the biomarkers studied here.

### 3.8 Health Economic Analysis

As shown below, Table 3 presents the cost comparison for a projected 10,000 patient cohort and the estimated costs: Universal PET ($30,000,000, 10,000 scans), Plasma + PET (gray zone; $18,317,000, 4,439 scans) which demonstrated 39% savings, Staged Algorithm ($9,880,800, 1,331 scans) which demonstrated 67% savings, Staged + MRI ($8,548,800, 887 scans) which resulted in 71% savings.

GRAD reduces per-patient costs from $3,000 to $855–988, with 86.7–91.1% reduction in PET utilization.

## 4. Clinical Application: Case Examples

### Case 1 - High Confidence Negative:

Individual in 60–64 age range with subjective cognitive decline. Plasma p-tau217 0.04 pg/mL, Gatekeeper probability 8%. Classification: Amyloid NEGATIVE (NPV 91.8%). Action: Reassurance, lifestyle modifications, routine follow-up. Outcome: $3,000 PET avoided.

### Case 2 - Gray Zone Resolved:

Individual in the 75–79 age range with MCI. Plasma p-tau217 0.12 pg/mL, Gatekeeper probability 45% (gray zone). Reflex panel: elevated GFAP, low Aβ42/40, hippocampal atrophy on MRI. Reflex probability: 78%. Classification: Amyloid POSITIVE. Action: Discuss anti-amyloid therapy eligibility.

### Case 3 - Persistent Uncertainty:

Individual in the 50–54 age range with atypical presentation and mixed vascular findings. Reflex probability: 52%. Classification: Indeterminate. Action: Recommend confirmatory PET. Outcome: GRAD appropriately identified cases requiring definitive imaging.

## 5. Discussion

### 5.1 Principal Findings

We developed a two-stage algorithm that provides classifications for the majority of patients while routing uncertain cases for additional workup. The Gatekeeper resolves 55.6% of cases with 88.8% accuracy using univariate p-tau217; for the remaining patients who fall into the gray zone, the Reflex classifier improves discrimination from chance to 69.7% accuracy. MRI integration provides statistically significant incremental value, and health economic modeling projects 67–71% cost reduction versus universal PET.

### 5.2 Performance by Design

The staged approach intentionally stratifies cases by diagnostic certainty rather than forcing classification through a single model. Stage 1 identifies cases where p-tau217 alone provides high confidence (88.8% accuracy, NPV 91.8%); Stage 2 addresses inherently difficult cases through multi-marker integration. This mirrors established laboratory medicine workflows, such as TSH with reflex free T4 for thyroid testing, antibody with reflex confirmatory for HIV screening, and PT/INR with reflex factor assays coagulation. Our framework essentially applies these proven paradigms to Alzheimer’s blood biomarkers.

### 5.3 Clinical Implementation

The 25%/75% thresholds balance resolution rate with accuracy. For anti-amyloid therapy eligibility (which have higher false-positive stakes), protocols should consider utilizing 20%/80% for higher specificity. For clinical trial screening (which have, in contrast, high false-negative stakes), consider 30%/70% for higher sensitivity.

The ΔAUC improvement (+0.025, p=0.014) and 2.5-fold variation in amyloid positivity by hippocampal tertile demonstrate that MRI, which is already routine in dementia evaluation, provides valuable independent information at no additional cost.

Beyond direct cost savings as demonstrated in this paper and improved diagnostic capabilities, staged testing improves geographic access to care by virtue of the fact that plasma testing is available at any equipped laboratory, in comparison to specialized PET centers. A movement towards this kind of testing will ultimately reduce patient burden, benefiting underserved and rural populations.

### 5.4 Limitations

ADNI participants are predominantly White (89%) and highly educated, and as such, validation in diverse populations is essential. A4’s amyloid-positive-only enrollment precluded traditional sensitivity/specificity. Cross-platform differences (Janssen/UPENN vs. Lilly assays) may introduce systematic variation. Lastly, the cross-sectional design precludes longitudinal assessment.

## 6. Conclusions

Our GRAD two-stage Gatekeeper-Reflex algorithm resolves diagnostic uncertainty in plasma p-tau217 interpretation by identifying high-confidence cases first and applying multi-marker classification to the remaining inherently uncertain cases. The framework mimics clinical laboratory testing and provides efficient, cost-effective diagnostic support (67–71% cost reduction vs. universal PET) while maintaining clinical accuracy. By design, our algorithm stratifies patients based on diagnostic certainty, offering a realistic pathway for implementing plasma biomarkers in clinical practice in the modern era of anti-amyloid Alzheimer’s therapies.

## Figures and Tables

**Figure 1.**
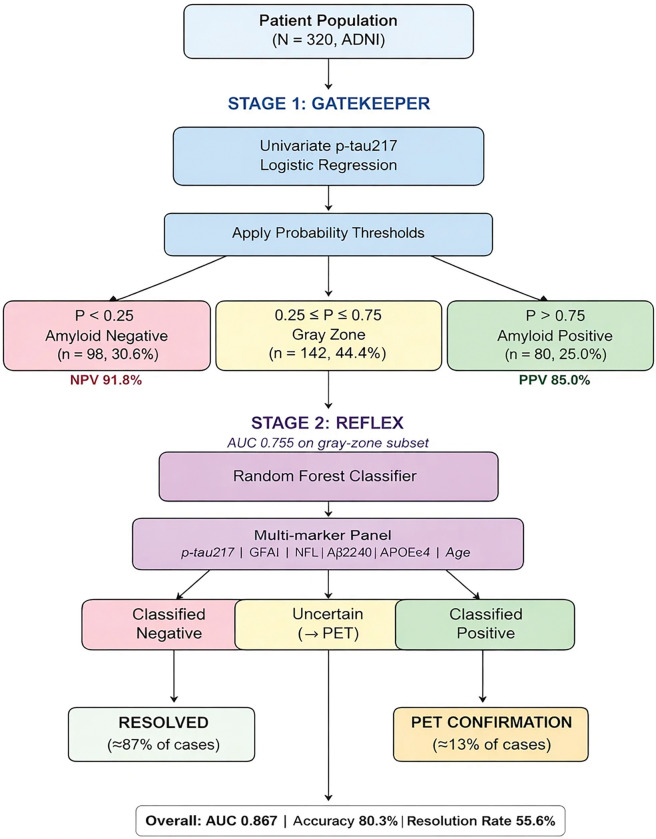

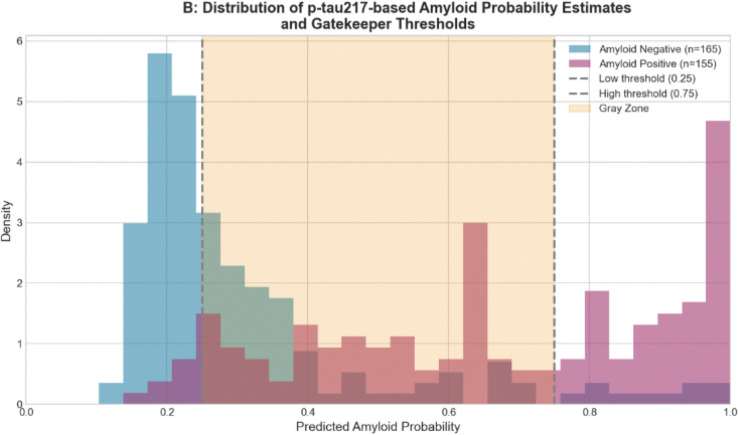
(A) Two-stage Gatekeeper-Reflex algorithm workflow showing patient flow from plasma testing through final classification. (B) Distribution of p-tau217-based amyloid probability estimates with Gatekeeper thresholds (25%/75%) showing classification zones: Amyloid Negative (P<0.25), Gray Zone (0.25 ≤ P ≤ 0.75), and Amyloid Positive (P > 0.75).

**Figure 2.**
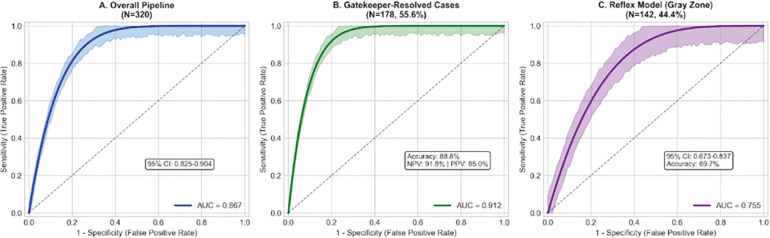
ROC curves for (A) overall pipeline (AUC=0.867), (B) Gatekeeper-resolved cases (AUC=0.912), and (C) Reflex model for gray zone (AUC=0.755). Shaded regions: 95% bootstrap CIs.

**Figure 3.**
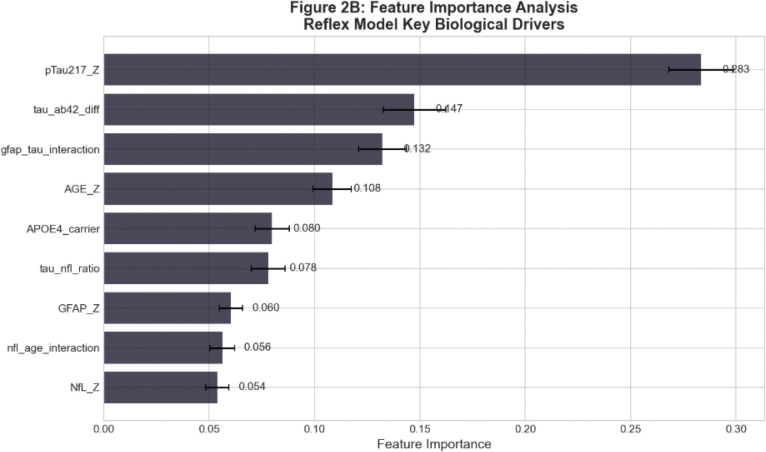
Feature importance from Reflex model showing contributions of primary biomarkers, engineered features, and demographic/genetic factors. The tau_ab42_diff, or difference in p-tau 217 and AB42/40 was correctly weighted as the highest feature in the diagnostic gray zone.

**Figure 4.**
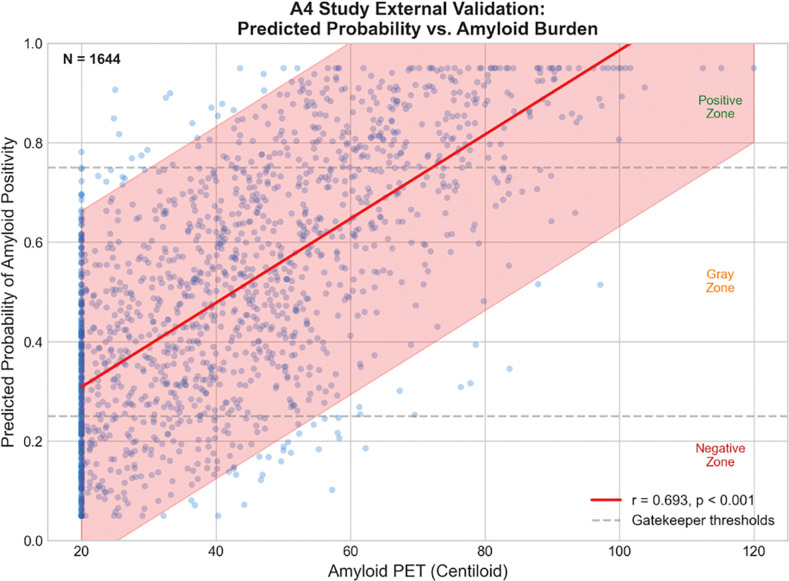
External validation: correlation between predicted probability and centiloid (r=0.693, p<0.001), demonstrating GRAD’s biological validity.

**Figure 5.**
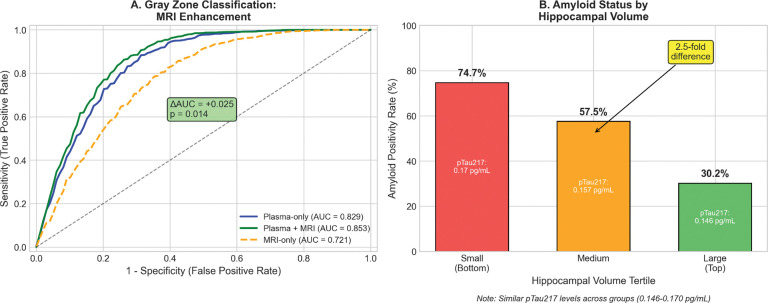
MRI enhancement: (A) ROC curves comparing plasma-only (AUC=0.829) vs. plasma+MRI (AUC=0.853); (B) Amyloid positivity by hippocampal volume tertile showing 2.5-fold variation.

**Figure 6.**
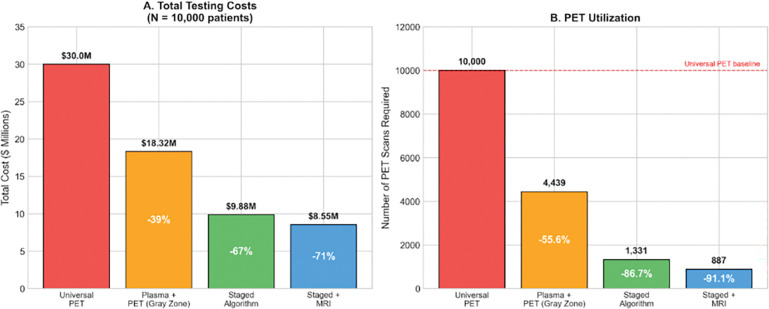
Health economic comparison across testing strategies for a projected 10,000-patient cohort. The GRAD staged algorithm excluding MRI integration scored the lowest cost, but required a higher PET utilization for indeterminate cases within the gray zone.

**Table 1.** Baseline Participant Characteristics

Characteristic	ADNI (N=320)	A4 (N=1,644)
**Age, years** (mean ± SD)	72.5 ± 6.8	71.8 ± 4.7
**Female**, n (%)	152 (47.5%)	953 (58.0%)
**Education, years** (mean ± SD)	16.3 ± 2.6	16.8 ± 2.4
**White race**, n (%)	285 (89.1%)	1,479 (89.9%)
**APOE ε4 carrier**, n (%)	113 (39.0%)	598 (36.4%)
**Amyloid positive**, n (%)	155 (48.4%)	1,644 (100%)
**p-tau217, pg/mL** (median [IQR])	0.110 [0.064–0.228]	0.152 [0.098–0.234]
**Cognitive status**: CN / MCI / AD	142 / 126 / 52	1,644 / 0 / 0

**Table 2.** Complete Pipeline Performance Metrics

Metric	Value	95% CI
**AUC**	0.867	[0.825–0.904]
**Accuracy**	80.3%	[75.9%–84.4%
**Sensitivity**	80.6%	[74.5%–86.6%]
**Specificity**	80.0%	[73.5%–85.4%]

**Table 3.** Cost-Impact Analysis by Diagnostic Method

Strategy	Total Cost	Cost/Patient	PET Scans Required	Savings vs. PET
**Universal PET**	$30,000,000	$3,000	10,000	Reference
**Plasma + PET (Gray Zone)**	$18,317,000	$1,832	4,439	39%
**Staged Algorithm**	$9,880,800	$988	1,331	67%
**Staged + MRI**	$8,548,800	$855	887	71%

## Research in Context

### Literature Review:

We searched PubMed, Google Scholar, and medRxiv databases for studies up to December 2025 that examined plasma p-tau217 diagnostic accuracy as well as “gray zone” management of patients. While several studies demonstrate area-under-the-curve (AUC) of >0.90, these studies largely compare cognitively normal individuals to those with established AD dementia with maximal biomarker separation [1–6]. The gray zone problem, affecting 30–50% of tested individuals, remains unaddressed in the vast majority of clinical implementation frameworks [7,8]. More recent work has established probability-based interpretation [9], but more cohesive algorithms for gray zone resolution through multi-marker integration remain rare if present. Furthermore, the health economic impacts of such resolution have not been fully established.

### Interpretation:

Our two-stage algorithm provides a workflow with clinical implementation potential, analogous to established laboratory medicine (i.e TSH with reflex free T4 testing). By first identifying high-confidence cases through univariate p-tau217 (55.6% resolution at 88.8% accuracy), and then applying multi-marker classification only to uncertain cases, we are able to achieve optimal resource utilization while simultaneously maintaining diagnostic accuracy. The finding that MRI usage provides statistically significant improvement (ΔAUC=+0.025) has practical implications given the fact that there is a reasonable level of MRI availability in clinical settings.

### Future Directions:

While this work accomplishes several key priorities, future work is required to validate them in diverse clinical populations. In addition, integration of other plasma markers (ex. MTBR-tau243), development of clinical decision support tools, reimbursement mechanisms, and longitudinal validation for treatment monitoring will be necessary to ensure the appropriate infrastructure exists to support providers and patients. Preliminary evidence suggests that %p-tau217 (the ratio of phosphorylated to total tau-217) and MTBR-tau243, a mass spectrometry-based marker of tau tangle pathology, may substantially improve gray zone classification by capturing complementary aspects of tau biology not reflected in absolute p-tau217 concentrations alone, which is a direction that future technical work should examine further.

## Highlights

- GRAD: Two-stage “Gatekeeper + Reflex for Alzheimer’s Disease” algorithm resolves indeterminate plasma p-tau217 or ‘gray zone’ patients with AUC of 0.755.
- Overall AUC of 0.867 (95% CI: 0.825–0.904) validated via leave-one-out cross-validation
- External validation in A4 Study demonstrates strong correlation with amyloid burden (r=0.693)
- MRI volumetric integration provides significant incremental value (ΔAUC=+0.025, p=0.014)
- Projected 67–71% cost reduction compared to universal PET screening
